# Finding the Keys to the CAR: Identifying Novel Target Antigens for T Cell Redirection Immunotherapies

**DOI:** 10.3390/ijms21020515

**Published:** 2020-01-14

**Authors:** Rebecca C. Abbott, Ryan S. Cross, Misty R. Jenkins

**Affiliations:** 1 Immunology Division, The Walter and Eliza Hall Institute of Medical Research, Parkville, VIC 3052, Australia; abbott.b@wehi.edu.au (R.C.A.); cross.r@wehi.edu.au (R.S.C.); 2 Institute for Molecular Science, La Trobe University, Bundoora, VIC 3086, Australia; 3 Department of Medical Biology, The University of Melbourne, Parkville, VIC 3052, Australia

**Keywords:** chimeric antigen receptor T cells (CAR T), Bi-specific T cell Engager (BiTE), immunotherapy, oncology, antigen selection, target antigen, proteomics, glycomics, lipidomics, antigenic screen, cell surface antigen, phage display

## Abstract

Oncology immunotherapy has been a significant advancement in cancer treatment and involves harnessing and redirecting a patient’s immune response towards their own tumour. Specific recognition and elimination of tumour cells was first proposed over a century ago with Paul Erlich’s ‘magic bullet’ theory of therapy. In the past decades, targeting cancer antigens by redirecting T cells with antibodies using either bispecific T cell engagers (BiTEs) or chimeric antigen receptor (CAR) T cell therapy has achieved impressive clinical responses. Despite recent successes in haematological cancers, linked to a high and uniformly expressed CD19 antigen, the efficacy of T cell therapies in solid cancers has been disappointing, in part due to antigen escape. Targeting heterogeneous solid tumours with T cell therapies will require the identification of novel tumour specific targets. These targets can be found among a range of cell-surface expressed antigens, including proteins, glycolipids or carbohydrates. In this review, we will introduce the current tumour target antigen classification, outline existing approaches to discover novel tumour target antigens and discuss considerations for future design of antibodies with a focus on their use in CAR T cells.

## 1. Introduction

High precision tumour targeting has been revolutionised by the emergence of T cell based immunotherapies utilising the infusion of activated, genetically engineered T cells, or by delivery of bispecific T cell engaging antibodies (BiTEs) [1]. Chimeric antigen receptor (CAR) T cells and BiTEs are the main forms of T cell redirection immunotherapies, using single chain variable fragment (scFv) targeting of tumours to induce target cell death. This approach has enabled the elimination of malignant cells, previously ‘invisible’ to the immune system, and provided excellent therapeutic results in patients with certain relapsed or refractory tumours. This occurs particularly efficiently in the case of CAR T cells, where the fusion of antibody binding domains to T cell signalling proteins such as CD3, has the capacity to redirect the T cell specificity for antigens. A major advantage of a CAR is that the T cells are activated and can exert effector functions such as release of cytotoxic granules and cytokines without recognition of peptide presentation by major histocompatibility complex (MHC) as the CAR interacts directly with cell surface molecules. 

**Figure 1:**
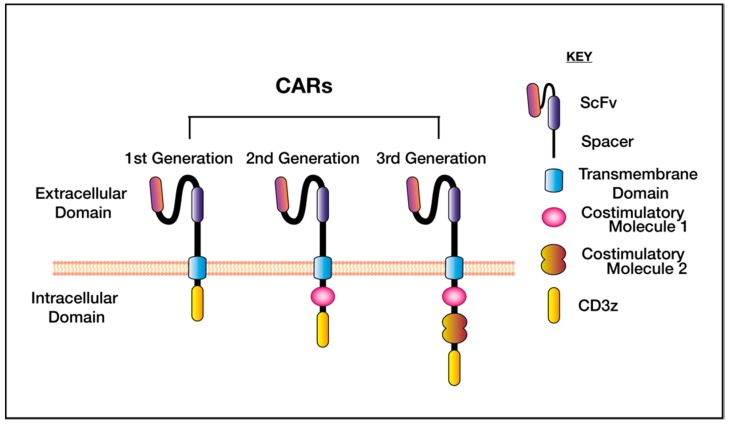
The generations of chimeric antigen receptors (CAR). The CAR designs differ based on the intracellular signalling tail. First generation CARs feature only the transmembrane domain fused to CD3ξ, these proliferated poorly in vivo. Second and third generation CARs differ in the inclusion of one (second generation) or two (third generation) costimulatory domains—these are commonly CD28 or CD137 (4-1BB).

Designed to mimic the functions of natural immune receptors, CAR T cells are a living drug, generated by introducing a synthetic receptor into patient’s autologous T cells, allowing CAR binding to tumour cells via an antibody binding domain, specific for the target antigen. The first CARs, as described by Eshhar in 1993 contained an scFv fused only to the CD3 complex [2]. These ‘first generation’ CAR T cells proliferated poorly and were unable to mediate complete tumour clearance [2], and subsequent designs featured fusion of the scFv to a T cell receptor (TCR) costimulatory domain, commonly CD28 [3,4] or CD137 (also known as 4-1BB) [5] endodomains (Figure 1). The CD3ξ signalling tail and incorporation of one or more costimulatory domains, bypasses the need for external primary and secondary activation signals, which initiate cytotoxicity and cytokine secretion upon T cell engagement. The design and protein engineering of CARs has evolved dramatically in recent years, involving variation in the ectodomain, transmembrane domain, linker and hinge regions, as summarised in [6]. The choice of co-stimulation has also been extensively reviewed [7,8].

**Figure 2:**
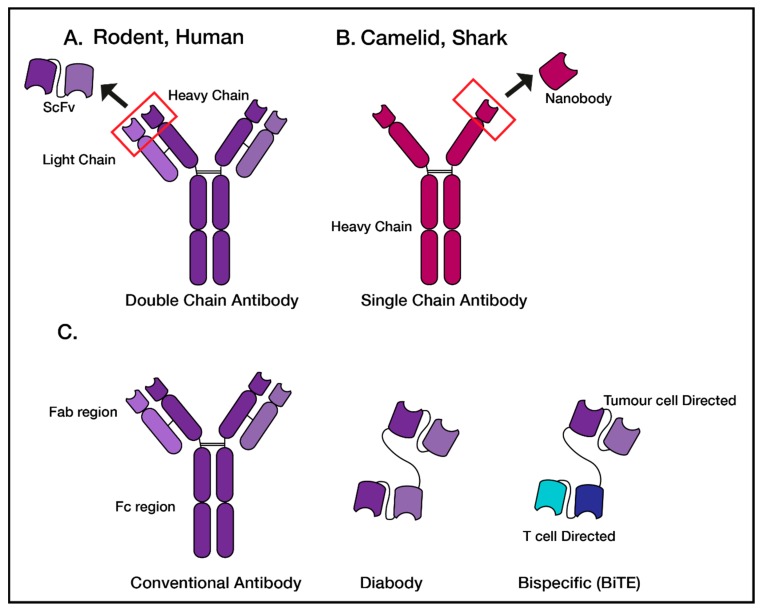
Common Antibody and antibody fragments which can be generated to validate target antigens. (**A**) Upon antigenic challenge, full sized dual chain antibodies are produced in model systems such as rodents and humans. The antibody fragment generated is a single chain variable fragment (scFv). (**B**) Camelids and sharks produce single, heavy chain only antibodies, with a nanobody antibody fragment. (**C**) The antibody fragments discussed in this review include diabodies—two fused scFvs or nanobodies of the same antibody, and bi-specific antibodies made of two fused scFvs with different specificities.

Bispecific T cell engagers are a fusion of two antibody binding domains, linked by a flexible linker sequence (Figure 2). Each arm of the BiTE displays a different specificity, with one arm to endogenous T cells (via CD3ε), and the second arm to a tumour antigen of choice. There are over 50 BiTEs in clinical trials for various malignancies, including CD19-targeted for acute lymphoblastic leukaemia [9], subsequently called Blinatumomab which was FDA approved in 2014 for the treatment of minimal residual disease in acute B cell lymphomas. 

Several trials have reported successful applications of CAR T cell therapy in haematological cancers, prominently the use of anti-CD19 CAR T cells in the treatment of leukaemia [10,11,12,13]. Targeting CD19 in leukaemia was successful in part due to the easy accessibility to leukemic targets and the homogenous expression of CD19 on the ‘dispensable’ B cell population. Despite several potential solid tumour targets being investigated, there has been limited reports of long term responses [14]. Both BiTEs and CAR T cells offer an exciting possibility to redirect a patient’s endogenous T cells to induce antitumour activity, and these two forms of immunotherapy have been recently excellently contrasted and reviewed [15].

Despite the ability to engineer, redirect and influence cell functions and interactions, there are challenges associated with targeting proteins expressed on tumour cells. Antigen escape and downregulation from the tumour cell surface are major obstacles in the clinic, limiting therapy effectiveness by leading to antigen negative relapse [14,16,17], and as reviewed in [18]. The lack of target antigen expression on every cell within a tumour and therefore the inability of T cell mediated therapies such as CARs or BiTEs to eliminate all malignant cells is the most likely mechanism behind antigen escape. Thus, providing the opportunity for antigen negative cells to remain and outgrow, forming target negative tumours at relapse. Additionally, selection pressure on malignant cells results in only the fittest tumour cells surviving, therefore, proteins which are not absolutely essential to tumour cell survival may be downregulated or lost from the cell surface. Stable homogenous tumour target antigen expression has been shown to result in more durable CAR T cell immunotherapy, with the most heterogeneous tumours undergoing antigen escape [13,19,20]. Antigen escape remains a major challenge to effective cell based therapies, and approaches such as dual antigen targeting have shown promising early results [21]. Systematic target antigen selection may be critical to overcoming the challenges associated with antigen escape, and treatment of solid tumours [22].

T cell redirection immunotherapies, such as CARs and BiTEs are capable of inducing a reduction in cancer burden—evident by the success in treating some forms of haematological cancers. However, a limiting factor for the wider application of CAR and BiTE immunotherapy is the relatively small number of known tumour antigens for targeting. Innovation in ‘omics’ technology and protein production platforms have been essential for progress in the rapid generation of antibody binding domains required for CAR or BiTE development.

In this review we outline the types of target antigens that can be targeted using antibodies, and the exciting advancements in technologies and phage display screening platforms which have enabled the identification of novel single chain antibodies. We discuss the current approaches used to identify novel targets for therapeutic development for T cell redirection immunotherapy.

## 2. Classification of Target Cancer Antigens 

### 2.1. The Ideal Therapeutic Cancer Antigen

To date, the field of immunotherapy has relied on developing therapies specific for known and validated targets, a strategy which has yet to lead to effective treatments for many solid tumour types. The identification of antigens expressed in heterogeneous solid cancers which are not also expressed on critical healthy tissues is a challenge for the field. The ideal cancer antigen would be exclusively expressed at the tumour cell surface, with cancer antigens encoded by mutated genes among the safest. The next ideal antigen is one that is shared with a nonessential tissue, with CD19 being the most recognisable in targeting of B cell malignancies. Two CD19 specific CAR T cell products were FDA approved in 2017, tisagenlecleucel (Kymriah) [23,24] for B cell precursor Acute Lymphoblastic Leukaemia (B-ALL) and axicabtagene ciloleucel (Yescarta) for large B cell lymphomas [25].

Tumour antigens have traditionally been largely restricted to cell surface expressed proteins, however, target antigens can also include protein post-translational modifications such as carbohydrate chains or even lipids. Antigens can be classified as either tumour specific antigen (TSA), tumour associated antigen (TAA) or cancer germline antigens (CGA), based on their expression patterns (Table 1).

### 2.2. Tumour Specific Antigens (TSA)

Tumour specific antigens (TSA) are exclusively expressed on malignant tumours. TSA are usually thought of in the context of mutations in proteins presented on the cell surface via MHC, such as IDH1 R132H presented in HLA-A2 on glioma cells [30]. However, the category of TSA can be expanded to include tumour specific glycosylation, such as TnMUC1 [31], tumour specific mutations in cell surface proteins, such as EGFRvIII [32] and misfolded proteins that escape refolding within the endoplasmic reticulum, such as misfolded-EGFR [33]. In glioblastoma, two clinical trials using EGFRvIII targeting CAR’s have recently been reported, the Novartis (scFv clone 2173) scFv is of second generation and designed with a CD137 costimulatory domain [26] and the NCI (scFv clone 139) CAR which incorporates a third generation signalling tail containing both CD28 and CD137 domains [19]. Maus and colleagues, using the 2173 CAR, have demonstrated successful CAR T trafficking to the brain following peripheral infusion, with no significant toxicities and CAR T cell persistence [34]. In contrast, the 139 scFv CAR T cell clinical trial resulted in dose limiting toxicity [19]. Whilst both trials were successful in meeting safety criteria, neither trial showed objective responses in secondary measures, despite presence of long-term CAR transcript [19]. These studies highlight the enhanced safety by targeting TSA, and provide impetus for further identification of TSA in other cancer types.

### 2.3. Tumour Associated Antigens (TAA)

Therapeutic targeting of tumour associated antigens (TAA) has been successful in some cases, but also served as a warning of the potential off tumour effects that can be associated with therapy. This class of targets are broadly defined as either having a greater level of expression in malignant tissue compared to matched healthy tissues, such as human epidermal growth factor receptor 2 (HER2), or are lineage restricted in their expression, such as CD19. Unlike TSA, TAA will often have on-target side effects which make it more difficult to disentangle direct treatment related side-effects, such as tumour lysis syndrome, with on-target/off-tumour toxicities. CD19 targeted CAR T cell therapy in B-ALL was the breakthrough therapy to show that CAR T cells could be clinically effective [35]. Expectedly, the lineage restriction of targeting CD19 results in B cell ablation, thus patients require ongoing administration of intravenous immunoglobulin [11] to prevent infection. Therefore, the targeting of lineage specific TAAs is possible, but only justified when the healthy tissue is considered to be dispensable or there is an acceptable level of toxicity.

Antigen load can also be used to discriminate a TAA. One of the best studied examples is HER2, an orphan growth receptor which is often overexpressed in epithelial tumours. The targeting of HER2 using monoclonal antibodies, such as Trastuzumab (Herceptin), has been highly successful in breast cancer [36], with few major side effects [37]. However, despite the success of Trastuzumab forming the foundation for a CAR T cell clinical trial, Rosenberg and colleagues reported a patient fatality after treatment with HER2-specific CAR T cells, due to on-target/off-tumour toxicity [29]. This 2010 report served as a cautionary tale to the field, however subsequent clinical trials with HER2-targeting CAR T cells have been conducted and have demonstrated safety with lower doses of CAR T cells [38], albeit coupled with a lack of efficacy, highlighting the need to accommodate antigen load with CAR T cell dose when developing T cell therapies which target TAA.

### 2.4. Cancer Germline Antigens

CGAs are a group of TAA which may make good immunotherapy targets due to their predominantly cancer restricted expression in adults and their high immunogenicity [39]. CGA expression is largely restricted to the testis or ovary, making them ideal targets due to restricted expression in adult somatic tissue, limiting on-target/off-tumour toxicities. Furthermore, there is evidence that hypomethylation of tumours induces increased expression of CGA [40]. The CGA IL-13Rα2 is expressed with high abundance on a majority of brain malignancies [41]. A CAR targeting IL-13Rα2 was recently reported to have shown signs of efficacy in the treatment of a single patient with multifocal Glioblastoma [14]. Initial regression of intracranial and spinal tumours was observed, and sustained for 7 months, before relapse. Treating heterogeneous solid tumours is proving a challenge, and both this single patient case study [14], as well as a study targeting EGFRvIII [34] have both demonstrated that CAR T cells can traffic to the solid tumour and cross the blood–brain barrier. Despite CAR T cell therapy increasing endogenous T cell recruitment to the tumour, CAR targeting of a single antigen alone has not shown to be sufficient or efficacious to overcome the problems of antigen escape, clinically. Therefore, novel targets are urgently required to widen the availability of treatment options.

## 3. Target Antigen Identification: From Screening to Validation

One of the major hurdles in immuno-oncology is the identification of appropriate targets that result in disease regression, whilst leaving healthy tissues unharmed. Thus far, the majority of scFvs in CAR T cell clinical development have been derived from pre-existing monoclonal antibodies [26,27]. Whilst this is a valid approach for the development of CAR T cells, for expansion of efficacy to new indications, novel CAR targets must be identified. Here we outline the current approaches to identifying novel antigens for tumour targeting, including their advantages, limitations and applicability.

### 3.1. Cell Surface Molecule Identification

The cell surface is covered in an array of molecules including lipids, glycolipids, proteins, and proteins with post-translational modifications, collectively referred to as the ‘surfaceome’ of the cell. A molecule in any of these biological classes has the potential to be a targetable antigen for therapy development, provided it fits the criteria of a target antigen for the particular tumour type and location in the body. The identification of surfaceome molecules differs between the biological classes, but commonly involves various forms of mass spectrometry (MS).

Proteins are the predominant class of target in cancer therapies and therefore identifying novel cell surface expressed proteins is of great interest. Combined efforts in the detection of cell surface proteins has led to the discovery of over a dozen therapeutic antibodies. Proteins expressed on the cell surface are collectively referred to as the ‘cell surface proteome’, and once defined, the surface proteome must be bioinformatically refined to identify potentially therapeutically relevant proteins. The techniques to identify cell surface proteins can be direct, such as cell surface capture proteomics or indirect such as using transcriptomics to predict membrane protein expression.

Cell surface proteomics is a collection of techniques which directly and selectively identifies the surface proteome. The analysis of the proteome relies on enrichment and capture or solubilisation of cell surface proteins before identification by MS. To do this there are a variety of methods available, such as ultracentrifugation, aminooxybiotinylation or silica bead coating [42]. These techniques each employ a different property of either the cell surface proteins themselves or that of the lipid membrane. However, due to the nature of MS, each of these approaches are biased towards the identification of the most abundant proteins, or those most conducive to purification, within a sample. Standard total proteomic approaches also often under-represent the surface proteome due to their lower expression and detection compared to intracellular proteins (recently reviewed in [42]). Constant advances in technology improve screening methodologies, and proteomics is a rapidly expanding discipline. In 2019, an improved method for identifying the surface proteome using chemical coupling and biotinylation was demonstrated using two breast cancer cell lines [43]. The authors report the new method is simpler, highly sensitive and has broad applicability including novel target discovery.

Another approach in the identification of the cell surface proteins is to use transcriptomic data to indirectly determine what is expressed on the cell surface based on expression of predicted transmembrane proteins. Whilst these techniques provide a greater number of targets compared to cell surface proteomics, proteins identified in transcriptomic screens may not be exclusively be expressed at the cell surface, and in fact there can be a low correlation between transcript and protein abundance in relation to cell surface proteins [44]. Bioinformatic approaches to identify proteins with transmembrane domains are employed, which can present a problem in that transmembrane domains are complex, varied in structure, and difficult to define (reviewed in [45]). The greatest limitation of transcriptomic approaches is in that transmembrane domains must be defined in order for computer algorithms to identify them [46], and only those which fit the given definition will be identified.

In addition to cell surface expressed proteins being attractive for immunotherapy targeting, they can also be useful to identify as biomarkers of disease. A screen for blood shed surface proteins was carried out by Ghosh and colleagues in 2017, to identify a serum biomarker panel for Glioblastoma (GBMSig) [47]. Ghosh and colleagues utilised an integrated pipeline of discovery proteomics, supported by transcriptomics to identify potential new targets. This study highlights the power of a combinatorial approach in filtering large protein lists to smaller, manageable data sets. Whilst combinatorial approaches have great potential, integration of ‘omics’ datasets remains difficult, with few published examples [48]. One recent example reported for Ovarian Cancer used shotgun proteomics (high performance liquid chromatography combined with mass spectrometry) on biobank tissue to identify chemotherapy sensitive novel biomarkers [49]. Phosphoproteomics was applied to further identify target protein function. Advances in sample fractionation methods and detection sensitivity enabled extraction of a large number of proteins from this biobanked tissue, showing this discovery workflow to be useful to not only identify targets, but also determine the protein function.

Direct methods of cell surface protein identification such as proteomics, or indirect methods such as transcriptional approaches are the first step towards therapeutic target identification. However, each method is not without its own advantages and disadvantages, highlighting the power of combination approaches, where both proteomic and transcriptional approaches can be combined to offset selection and identification bias.

Glycoproteins are another class of cell surface molecules which may be suitable therapeutic targets. As reviewed by Arub Everest-Dass, there are three main approaches to studying glycosylation [50]. The first is characterisation of intact glycoproteins. This is performed most commonly by different forms of MS. However, the presence of carbohydrate groups decreases the efficiency of ionisation. Characterisation of protease-digested glycopeptides is a second approach. The enzymatic digestion of glycoproteins to peptides overcomes the issue of mass limited resolution of intact glycoprotein analysis by MS, however, glycopeptides are detected with a low signal by MS and therefore require enrichment before or during analysis [50]. The structural analysis of N- and O-glycans released from proteins is currently the best approach in investigating the structure, sequence, branching and linkage of glycans. The recent advances in technology to determine these structures is detailed [50], though two key methodologies include glycan MS fragmentation spectra to determine glycan structure and Ion mobility to identify glycans from complex mixtures.

Surface expressed lipids may also present as valid targets for targeted treatment. The study of lipidomics is the profiling and quantification of lipid molecules and relation to biological function. This field has been advanced through improvements in technologies such as Nuclear Magnetic Resonance spectroscopy, chromatography but most significantly, mass spectrometry. Liquid chromatography mass spectrometry (LC-MS) is the most common technique used for lipidomics research, due to the high sensitivity and specificity, and separation efficiency of this method [51]. MS based lipidomics can be divided into two subgroups—nontargeted lipidomics, which is more relevant for this review, is used to identify the lipid molecules in a system. Contrastingly, targeted lipidomics is applied to characterise specific lipid molecules. The technological and protocol advances in lipidomics have been recently described [51].

From the list of surface molecules, the next stage in target identification is refinement of the identified molecules to those which are most likely to be clinically relevant.

### 3.2. Refinement of Target Antigen Pool

Depending on the cell type and method used, the number of cell surface expressed molecules can vary. A recent study using machine learning mapped the human surfaceome and identified up to 2886 proteins expressed at the cell surface, which can be interrogated here (wlab.ethz.ch/surfaceome) [52]. Furthermore, depending on the type of bioinformatic analysis, it may not be possible to determine the abundance of protein expression. Once the surface proteome has been identified, the next challenge is to refine the large list of proteins to those which are likely to be appropriate to target therapeutically. This is performed by comparing the proteome list to matched healthy tissues or pre-existing protein databases.

This second stage of target antigen selection requires filtering of the proteome dataset, with the type of desired target antigen, TSA, TAA or GSA, needing to be chosen as each will require a different analysis strategy. Resources such as the human protein atlas help in the refinement of analysis strategies by providing a compiled database of information regarding protein expression on a range of healthy and malignant tissue types [53]. Another useful resource is the Universal Protein Knowledgebase (UniProt)—a mergence of three databases which provides users with annotated protein sequences [54] (www.uniprot.org). To complement the UniProt database, neXtProt is a free database which also contains information regarding annotated protein sequences, though with a focus on human proteins [55] (www.nextprot.org). Finally, Human Proteinpedia is another searchable database for proteins, though differs from other databases in that the information is collated from proteomic analysis [56]. However, this database is incomplete and comparing between proteomic runs and technology platforms has inherent difficulties.

These resources have been generated to enable searching for specific protein expression on tissues throughout the body, with protein expression validated by antibody staining.

The filtering of glycomic datasets can be performed with a database similar to the UniProt system. UniCarbKB is an online database in which glycomics research is collated and annotated (http://www.unicarbkb.org) [57]. The field of lipidomics is not as advanced as that of proteomics, and therefore, there are multiple databases of identified and theoretical lipids, each with their advantages and disadvantages [58]. In 2013, a new database ‘LipidHome’ was established to fill the gaps in the existing lipid databases, with a clear distinction between experimentally validated and theoretical lipids (http://www.ebi.ac.uk/apweiler-srv/lipidhome) [58].

These databases allow for validation of tissue expression and can be used to cross reference to eliminate molecules expressed on healthy tissue. However, in the discovery of novel therapeutic targets there will be a long list of molecules with unknown biology, and no antibodies, which limits these databases in helping to validate whether such molecules are viable targets. Therefore, a potential better application of these databases is to cross reference the molecules identified in target discovery to remove any molecules already identified in these databases. This will enable the narrowing down of the list to completely novel targets, which will require novel tool discoveries outlined below for validation.

### 3.3. Generation of Antibodies for Therapeutic Screening

There are a multitude of tools which can be generated to examine and validate a selected protein as a therapeutic target. These tools are commonly full-sized antibodies, or antibody fragments, which will differ in structure and function, depending on the model system which they were produced in. Conventional antibodies consist of a dual heavy and light chain design, containing constant and variable regions, with specificity due to complementarity determining regions (CDR loops). This dual chain antibody structure is shared by rodents and humans, whereas camelids and sharks produce single heavy chain antibodies ((Figure 2) and reviewed in [59]).

Full length antibodies can be broken down into Fab target binding domains and Fc immune receptor binding domains. The Fab of an antibody, lacking the Fc domains, can be used to generate short chain variable fragments (scFvs), composed of the variable heavy and variable light domains linked using a short flexible peptide or a nanobody, which is comprised of the single variable heavy domain from a camelid or shark [60]. Once in the scFv or nanobody format, different therapeutic modalities can be tested such as diabodies, BiTEs or CARs. These engineered fragments have been developed as therapeutics on their own, and many are approved or in clinical development [59].

There are two main screening methods to identify antibodies or antibody fragments (binders) to target antigens, either generating antibodies in animals or display library platforms, such as bacteriophage display.

The main screening method uses immune-competent animals in the process for generating monoclonal antibodies, and has been condensed into five steps in Monoclonal Antibodies: A Tool in Clinical Research [61]. In summary, the antigen of interest must be purified before being injected into the animal. This elicits an immune response, with then isolation of B cells from either blood or lymph tissues and fusion to a specific type of myeloma cell—thereby immortalising the B cell in a hybridoma. The process to generate hybridomas was first described in 1975 by Kohler and Milstein [62]. Once a hybridoma has been generated, the antibodies secreted by the hybridoma can be screened for specificity and affinity. Recent advancements in genome editing has revolutionised the field, such as the replacement of endogenous mouse antibody gene with that of human origin (reviewed in [63]). This technology has generated two strains of humanised mice; HK and HL [64], whereby upon antigen challenge, the B cells from these mice produce a human antibody [64]. Though the diversity of the antibodies generated is limited to that of the human donor, this technology eliminates the arduous process of humanising and affinity tuning murine antibodies. The first process of humanising antibodies came after Neuberger et al.’s landmark paper in which manipulated immunoglobulin genes (Fc) were first introduced into lymphocytes to produce novel antibodies [65]. This was followed by the humanising of phage display for therapeutic use by Greg Winter and colleagues [66].

Whilst traditional methods of developing monoclonal antibodies have relied on the immunisation of animals in the past 20 years, powerful modern screening platforms have evolved to rapidly develop protocols enabling the identification of high affinity lead immunotherapy targets. Such platforms include display libraries which are usually composed of bacteriophages, but can include yeast libraries as well. These are all in vitro screens performed at the bench, with the display libraries containing either single or dual domain antibodies. The process of display screening has been reviewed in detail [67], and involves antibody fragments being used to coat phage proteins which are then used to infect *Escherichia coli*, which in turn assemble into antibody-displaying particles. These particles enter multiple rounds of screening to select those which bind to the target antigen. Phage display screening (bacterial or yeast) has been used to identify scFvs for immunotherapy development. These scFvs can be specific for whole proteins on the cell surface [68,69,70,71,72] or, with greater complexity, peptide:MHC complexes [73,74].

Once specific binders have been identified, regardless of the screening method, the pool of potential binders can be narrowed by analysing the structure, and further affinity tuning [75,76] and humanisation of the antibody scaffold [77,78] to gain the desired properties. When considering in vivo screening methods (using live animals), it is important to acknowledge affinity maturation can still be achieved in vivo with prime-boost strategies. Furthermore, if in vivo strategies fail to produce CDR loops with desirable qualities, it is possible to perform affinity tuning or maturation to alter the strength of scFv binding to the target antigen. A seminal study by Liu, June, Zhao and colleagues demonstrated that lower affinity CARs were capable of inducing greater levels of target cell death than higher affinity CARs. Critically, these lower affinity CARs demonstrated a greater capacity to distinguish between overexpressed and physiological levels of antigen expression compared to higher affinity CAR T cells [75]. This held true for both HER2 and EGFR directed scFvs. However, in vivo screening may result in the potential deletion of reactive binders during tolerance if the target antigen has a high homology to the animal being used to generate the antibodies.

In summary, in vivo screening allows for processes such as affinity tuning to occur more rapidly than ex vivo modifications, however, the pool of potential binders may be narrowed as a result of the potential deletion of reactive CDR binders in tolerance if the target antigen has a high homology to the animal being used to generate the antibodies. The advantage of in vitro screening is the provision of a larger pool of identified CDR’s, however this larger pool of CDRs requires greater effort to find binders to a given target.

### 3.4. Validation of Tools and Target

The final stage of novel antigen discovery is arguably the most revealing. It is essential that the antitumour antibodies or antibody fragments produced during the screening are functionally examined to evaluate safety and efficacy. Testing the new tool for binding specificity typically involves a number of in vitro approaches. Depending on antigen epitope conformation, antibody specificity can be validated by immunohistochemistry or Western blot using samples of healthy and malignant tissue [79]. A more stringent test is to use the binder in flow cytometry on live cells, as this is a greater approximation of the context in which the binder will recognise antigen. However, this method requires access to malignant and healthy tissue, which is not always practical or possible. Therefore, overexpression of target antigen on cell lines, or deletion from high expressing cell lines, is a common screening tool used to validate specific binding to target antigen. Once the tool is validated for its specificity, therapeutic efficacy can be investigated.

To test the therapeutic efficacy, binders can be engineered into BiTEs or CARs and examined in both in vitro and in vivo assays. Both CAR and BiTE development will require the isolation and use of T cells from healthy human donors. In the case of CARs, the T cells are transduced with a CAR using one of a variety of methods, critical to ensuring that the CAR can be produced and trafficked to the cell surface. BiTEs only require coculture with human T cells. In vitro studies commonly rely on the use of fluorescent and protein-based tags in the construct (such as Myc-tag or FLAG) to enable direct quantification of cell surface expression. There are different methods used for CAR transduction into primary cells using either retroviral [27], lentiviral [80] or transposon [81] approaches. The insertion of the CAR into T cells via mRNA is also possible, however, this method results in transient expression of the CAR and therefore multiple infusions of CAR T cells produced in this way are required to treat patients using this product. However, recent research has shown that the insertion of the CAR using purified mRNA produces CAR T cells with improved cytotoxic capacity compared to nonpurified mRNA CAR T cells, lower expression of checkpoint inhibitor molecules such as PD-1 and Lag-3, and improved in vivo function compared to non mRNA purified CAR T cells [82].

Once CAR expression at the cell surface is validated, CAR T cells and BiTE coculture are examined for therapeutic efficacy including cytotoxicity and cytokine secretion assays. To ensure safety, robustness and efficacy against tumours, T cell redirection immunotherapies are commonly performed in immunocompromised mouse models transplanted with xenografts, and therefore a key hurdle in the field is the availability of immunocompetent preclinical mouse models that recapitulate human disease. An ongoing challenge for the development of novel T cell redirection immunotherapy for human protein targets, is that the species mismatch can commonly prevent a full safety profile from being explored.

## 4. Conclusions and Perspective

New treatments for cancer—particularly rare and aggressive malignancies—are urgently required. Current approaches such as chemotherapy and radiation have long lasting systemic adverse effects for patients. Novel, personalised treatment approaches such as immunotherapy offer the advantage of specifically targeting cancer cells, however one of the major hurdles to the implementation of immunotherapy is the identification of new targets to widen therapeutic clinical options. Advances in proteomics, transcriptomics and bioinformatics outlined in this review offer a path forward for the discovery of novel targets for use in immunotherapy. These advancements when combined with advances in genetic engineering technologies will hopefully lead to the development of tumour specific therapies.

To effectively treat the diverse multitude of human cancers, many novel and specific target antigens must be discovered. The improvement and refinement of protein identification methods is contributing to the increasing popularity of cell surface proteomics. Therefore, it is easy to imagine that future breakthroughs in the identification of post-translational modifications to cell surface proteins will allow for a kaleidoscope of novel targets to be identified. This identification of more, and diverse, targets is of critical importance given the high rates of antigen escape, particularly in solid tumours. It is unlikely that malignant cells will be completely eliminated with a single targeted agent, and therefore multifactorial combination therapy—surgery, chemotherapy, radiotherapy and immunotherapy—will be required to provide patients with robust therapies. However, finding the key to unlocking the potency of T cell therapies is the first critical step.

## Figures and Tables

**Table 1:** The advantages and disadvantages of targeting three main types of target antigens; tumour specific, tumour associated and cancer germline antigens.

Type of Antigen	Expression	Advantage	Disadvantage	Example
Tumour Specific	Expressed only on malignant tissue	Unlikely to cause on target off tumour effects	Difficult to identify tumour specific targets—limited pool of possible proteins. Tumour heterogeneity between patients may make pan marker identification difficult.	EGFRvIII [26,27]
Tumour Associated	Expressed at low levels on healthy tissue or organs, expressed at a higher level on malignant tissue.	Increased likelihood of target identification	Possibility to cause on target off tumour effects. Extensive preclinical validation required to determine safety.	HER2 [28,29]
Embryonic Cancer Germline Antigen	Frequent expression on malignant cells, expressed on embryonic tissues and minimal expression on healthy adult tissue.	Regulators of many important cellular function pathways. Minimal expression on healthy tissue indicates this class of antigens may be potentially safer to target therapeutically.	Application of CARs targeting these antigens could result in the destruction of healthy reproductive tissue. Limited pool of antigens.	IL-13Rα2 [14]

## Abbreviations

CAR	Chimeric antigen receptor
BiTE	Bispecific T cell engager
scFv	Single chain variable fragment
HER2	Human epidermal growth factor receptor 2
EGFRvIII	Human epidermal growth factor receptor variant III
MHC	Major Histocompatibility Complex
MS	Mass spectrometry
CDR	Complementarity determining region
CGA	Cancer germline antigen
TAA	Tumour associated antigen
TCR	T cell receptor
TSA	Tumour specific antigen
